# Phytotoxicity effect of a highly toxic isolate of *Alternaria alternata* metabolites from Iran

**DOI:** 10.1016/j.toxcx.2024.100186

**Published:** 2024-02-15

**Authors:** Atefeh Sedighi, Abbas Mohammadi

**Affiliations:** Dept. of Plant Protection, College of Agriculture, Univerity of Birjand, Birjand, Iran

**Keywords:** Detached leaves, Inoculation, Pelargonium, Phytotoxic, Toxin

## Abstract

*Alternaria* species produce several mycotoxins, such as alternariol (AOH), alternariol monomethyl ether (AME), altenuene (ALT), altertoxin (ATX), tentoxin (TTX) and tenuazonic acid (TeA). This research aimed to isolate and identify mycotoxins from highly toxic *Alternaria alternata* (w19) and *A*. *tennuisima* isolates and their phytotoxicity effects. Fungal metabolites were extracted from 21-day cultures of *Alternaria* in a Czapek broth medium with the organic solvent chloroform/acetone and identified using the HPLC method. *Alternaria* metabolites were infiltrated *in vivo* into several plant leaves for phytotoxicity detection. The study investigated the impact of temperature, time, and metabolite concentration on phytotoxicity using the detached leaf infiltration technique. Five mycotoxins (TTX, TeA, ALT, AOH, and AME) were detected in *A. alternata* W19 isolate with 959.24, 102.03, 24.01, 9.04, and 2.44 ppm, respectively. *A. tennuisima* produce these toxins in a lower concentration. Infiltration of fungal metabolites induced leaf chlorosis and necrosis, which differs based on temperature, concentration and plant species. Based on our knowledge, this is the first report of *Alternaria* mycotoxins in Iran and a highly toxic isolate of *A. alternata* with rapid phytotoxicity on a wide range of susceptible hosts.

## Highlights

• Five mycotoxins of *Alternaria* isolates are reported from Iran.
• High toxic isolates of *Alternaria* induce fast necrosis and chlorosis in a wide range of plants.
• Pathogenicity of *Alternaria* isolates is changed by mycotoxins concentration.

## 1 Introduction

*Alternaria* species with global distribution and particular economic importance, produce more than 70 secondary toxic metabolites and mycotoxins, such as alternariol (AOH), alternariol monomethyl ether (AME), altenuene (ALT), altertoxin (ATX), tentoxin (TTX) and tenuazonic acid (TeA) mycotoxins (Aichinger et al., 2021; Solfrizzo, 2017; You et al., 2023). AOH, AME and TeA are produced by *Alternaria alternata, A. cucumeria, A. dauci, A. macrospora, A. porri, A. sesami, A. solani, A. tagetica* and *A. zinnia* species. These toxins were detected in apple, mandarin, olive, red pepper, tomato, sunflower seed, sorghum and wheat (Nagda and Meena, 2024). AOH and AME are mutagenic mycotoxins (Nagda and Meena, 2024). TeA, which inhibits *Mycobacterium tuberculosis* growth, is an active metabolite of *A. alternata* with higher toxicity than ALT, AOH, and AME (Sonaimuthu et al., 2011). TeA is toxic for humans, mice, poultry, and dogs, with mutagenic and genotoxic effects (Gambacorta et al., 2019; Nagda and Meena, 2024). This toxin degrades thylakoids or inhibits protein biosynthesis by ribosomes and photophosphorylation at the QB level (Liu et al., 2007). Other fungal species such as *Pyricularia oryzae* and *P. sorghina* produce TeA (Motoyama, 2020). ATX I and ATX II are secreted into infected apples, sorghum, and wheat by *A. alternata, A. tenuissima* and several other *Alternaria* species (Moretti and Sarrocco, 2016). ATXs are mutagenic and cause acute toxicity in mice (Dall’Asta et al., 2014). TeA, TTX, and maculosin are biological herbicides that affect chlorophyll accumulation, inhibit chloroplast development and induce leaf chlorosis (Sanodiya et al., 2010; Sotelo-Cerón et al., 2023). UV converts tentoxin to isotentoxin, which has more toxicity effects on *Galium aparine* weed (Lou et al., 2013).

We studied the effect of metabolites of some *Alternaria* isolates on *Pelargonium* and the metabolites of one isolate of *A. alternata* (W19), which induced necrosis of the *Pelargonium* leaves in a very short time. This study investigated the toxin production by this highly toxic isolate and its effect on different plants.

## 2 Materials and methods

*Alternaria* isolates were prepared from the fungal culture collection of the plant pathology laboratory at the University of Birjand, which had been collected from Birjand in South Khorasan province (Eastern Iran) during 2014–2016. The pathogenicity of these isolates has already been detected on several plants (Hossainnia, 2017). Ninety-four isolates of *Alternaria* were studied for phytotoxic metabolite production during this research. Secondary metabolite production and extraction were carried out from a 21-day culture of *Alternaria* isolates in a Czapek broth medium (Siciliano et al., 2015). Fungal tissue was removed using filter paper and centrifugation. Phytotoxic metabolites were extracted with an organic solvent consisting of chloroform and acetone in a ratio of 88:12 (Meena et al., 2017; You et al., 2023). The organic solvents were separated. After filtration with 0.45 μm syringe filters, they were evaporated. The sediments were then dissolved in distilled water or methanol and stored at −20 °C temperature.

### 2.1 The effect of *Alternaria* secondary metabolites on different plants

*Alternaria* metabolites were infiltrated into the *Pelargonium hortorum* and tomato leaves during the preliminary experiments. One milliliter from the extraction of 50 ml of Czapek broth media, which dissolved in 2 ml of distilled water, was infiltrated into the plant leaves using 5 ml syringes. Its effects on the leaf were investigated in less than 24 h. This method was used to study the effect of *Alternaria* metabolites on other plants during this research (Jones and Perez, 2022).

In the field and garden conditions, *Alternaria* metabolites were infiltrated into the *Panicum miliaceum*, *Avena sativa*, *Satureja hortensis*, *Carthamus tinctorius*, *Convolvulus arvensis*, *Acroptilon repens*, *Fraxinus excelsior*, *Cercis siliquastrum*, *Rudbeckia hirta*, *Melissa officinalis*, *Morus alba*, *Cydonia oblonga*, *Solanum lycopersicum*, *Punica granatum*, *Berberis thunbergii*, *Crataegus macrosperm*, *Phaseolus vulgaris* and *Alhagi maurorum* leaves (Jones and Perez, 2022; Meena et al., 2017). Extract of non-inoculated Czapek broth media was infiltrated into the *Pelargonium* leaves as the control.

### 2.2 The effect of temperature and concentration on metabolites phytotoxicity

*In three replications, pelargonium detached leaves responses to A. alternata (W19) metabolites were investigated at five temperatures (4, 10, 20, 25 and 40 ͦC)*. Detached *Pelargonium* leaves (sterilized by 10% sodium hypochlorite for 60 s) were placed into the sterile petri dishes and the moisture content was provided with sterilized distilled water. The different concentrations of metabolites (from 2 up to 100 percent in distilled water) from a Czapek culture media were infiltrated into a detached *Pelargonium* leaf with three replications (Jones and Perez, 2022; Meena et al., 2017).

### 2.3 Toxin identification

Toxins of three different isolates of *Alternaria* and Czapek broth culture medium as the control was purified by thin-layer chromatography (TLC) using chloroform/acetone (97:3,v:v) as a solvent system by TLC-UV based on the Hasan method (Hasan, 1995) and identified with HPLC (Solfrizzo et al., 2019) by professor Michele Solfrizzo in Italian National Council of Research.

### 2.4 Pathogenicity test

Pathogenicity test was performed based on Koch's principles (Wang et al., 2010) with inoculation of detached *Pelargonium* leaves with the 7-day-old colony of *Alternaria* on PDA. *Pelargonium* leaves were sterilized with 0.5 % sodium hypochlorite, placed on wet paper in sterile 9 cm Petri dishes, and inoculated with W19 (*A. alternata*), H4 (A. tenuissima), Z81 (Alternaria sp.), and control (PDA media). Pathogenicity and the development of symptoms were investigated after four days (Fontaine et al., 2021).

## 3 Results

The metabolites were infiltrated in *young Pelargonium leaves, especially the first and second leaves, which were* the most suitable for infiltration. The third leaf had no problem with infiltration, but it was not suitable due to metabolism reduction, leaf tissue changes due to environmental factors, and aging.

**Fig. 1:**
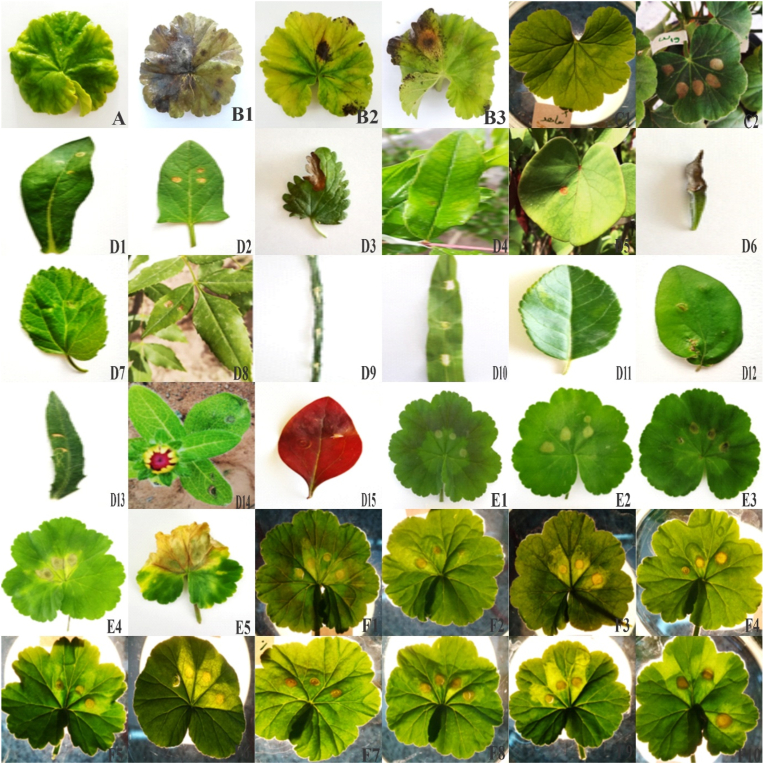
Effects of *Alternaria* metabolites on plant leaves. A1- A4 (Pathogenicity on *Pelargonium* detached leaves: control, W19, Z81 and H4), B1–B2 (*Pelargonium* leaves infiltrated by Czapeck and W19 extract), D1- D15 (infiltrated leaves of: *Convolvulus arvensis, Melissa officinalis, Punica granatum, Cercis siliquastrum, Satureja hortensis, Morus alba, Carthamus tinctorius, Fraxinus excelsior, Acroptilon repens, Avena sativa, Panicum miliaceum, Crataegus macrosperm, Cydonia oblonga, Rudbeckia hirta, Berberis thunbergii*), E1-E5 (effect of temperature on metabolites effect on *Pelargonium* leaves: 4, 10, 20, 25 and 40 C), F1–F10 (effect of metabolites concentration effect on *Pelargonium* leaves:(10, 20, 30, 40, 50, 60, 70, 80, 90, 100 percent).

In the primary test, three reaction types were observed on *Pelargonium* and Tomato leaves after inoculation with fungal metabolites. No changes were observed in the control and the first reaction type on *Pelargonium* and tomato leaves (Fig. 1C1-C2). The symptoms were observed in the second type in less than 12 h but in the third type after four days to 1 week. The necrosed regions were also different. Some isolates caused developed necrosis or chlorosis, but in the others, the symptoms were less developed and limited to the toxin infiltration site. An area of about 0.5 cm was necrotic in the infiltration region, but the other parts were yellowish and transparent.

Metabolites of *A. alternata* (W19) caused water soaking and tissue death in less than 24 h. The tissues were water-soaked on the first day, then became necrotic and dried. W19 isolate was selected for this research due to the high-speed rate of symptom incidence. Preliminary tests were performed on tomatoe, bean and *Pelargonium* plants. W19 isolate on tomato produced only leaf yellowing but beans showed tissue necrosis less than 24 h.

Because the intra-leaf distribution of the infiltrated solution was limited to the leaf vein, the chlorosis and necrosis were limited to the leaf vein.

### 3.1 The effect of the toxin on different plants

Infiltration into *M. officinalis* and *S. lycopersicum* tissues was easily done. Infiltration into *P. miliaceum*, *A. sativa* and *S. hortensis* was easier than infiltration into *C. tinctorius*, *C. arvensis*, *A. repens*, *F. excelsior* and *C. siliquastrum* leaves. Infiltration into the leaves of the other three plants, especially R. hirta, was very difficult due to the fluffiness of the leaves.

The infiltrated leaves of *P. miliaceum*, *A. sativa*, *S. hortensis*, *C. tinctorius*, *C. arvensis, A. repens*, *F. excelsior* and *C. siliquastrum* showed necrosis and tissue death. The symptoms in *P. miliaceum* and *A. sativa* occurred a few hours after infiltration as water soaking (Fig. 1 D1-D15).

*R. hirta*, *M. officinalis*, *M. alba*, and *C. oblonga* showed chlorosis and necrosis. In *M. alba* and *R. hirta*, yellowing was observed in the leaf vein. Necrosis of *M. alba* was less intense, but in *M. officinalis* was more intense.

*S. lycopersicum*, *P. granatum*, *B. thunbergii,* and *C. macrosperm* showed leaf yellowness at the toxin infiltration tissues. Leaves of *B. thunbergii*, *P. granatum*, as well as *C. macrosperm* showed clear hallo that were more pronounced under the light. In *P. vulgaris* and *Alhagi* leaves despite the easy infiltration of the metabolite into the leaf tissues, the result of infiltration was water soaking without chlorosis or necrosis (Fig. 1 D1-D15, Table 1).

**Table 1:** Time and type of plant reaction to *Alternaria alternata* W19 metabolites.

Common name	Scientific name	Plant Family	Symptoms	Times of Reaction(h)
Geranium	*Pelargonium hortorum*	Geraniaceae	Necrosis	Less then 24
Common millet	*Panicum miliaceum*	Gramineae	Necrosis	8
Oat	*Avena sativa*	Gramineae	Necrosis	8
Savory	*Satureja hortensis*	Lamiaceae	Necrosis	Less then 12
Black-eyed Susan	*Rudbeckia hirta*	Asteraceae	Chlorosis, Necrosis	Less then 12
Lemon balm	*Melissa officinalis*	Lamiaceae	Chlorosis, Necrosis	Less then 12
Ash	*Fraxinus excelsior*	Oleaceae	Necrosis	48
*Tomato*	*Solanum lycopersicum*	Solanaceae	Chlorosis	24
Common bean	*Phaseolus vulgaris*	Leguminosae	Water soaking	24
*Alhagi*	*Alhagi graecorum*	Fabaceae	No.	___
White mulberry	*Morus alba*	Moraceae	Chlorosis, Necrosis	12
Quince	*Cydonia oblonga*	Rosaceae	Chlorosis, Necrosis	12
Pomegranate	*Punica granatum*	Punicaceae	Chlorosis	12
Safflower	*Carthamus tinctorius*	Asteraceae	Necrosis	12
Japanese barberry	*Berberis thunbergii*	Berberidaceae	Chlorosis	48
Hawthorun	*Crataegus macrosperm*	Rosaceae	Chlorosis	48
Field bindweed	*Convolvulus arvensis*	Convolvulaceae	Necrosis	12
Leuzea repens	*Acroptilon repens*	Asteraceae	Necrosis	12
Judas tree	*Cercis siliquastrum*	Legominoseae	Necrosis	48

### 3.2 The effect of temperature on the incidence of toxin infiltration symptoms

The infiltration site was clear in the early hours of toxin infiltration at 4, 10, 20, and 25 °C. Three days after infiltration at 4 and 10 °C, *Pelargonium* leaf showed necrosis while at 20 °C occurred less than 24 h (Fig. 1 E1-E5).

At 25 °C, the infiltrated region was brighter than the other parts of the leaf in the first hour and necrosed after 8 h, but at 40 °C, necrosis occurred during the first hour after infiltration. The results showed that the toxicity of metabolites and tissue degradation directly correlated with environment temperature. This effect may be due to the higher toxicity of metabolites or increasing biochemical activities at high temperatures, which results in rapid death and faster destruction of plant cells.

### 3.3 The effect of toxin concentration on tissues’ necrosis

Different concentrations of infiltrated metabolites caused identical symptoms. At first, the infiltrated tissues showed brightness; after that, depending on the toxin concentration (from high to low), the time of symptom incidence was also increased. Severity and symptom incidence rates differed based on toxins concentration. Seventy or higher concentrations of metabolites caused necrosis within 4 h, but in concentrations of 40–60%, the incidence of necrosis symptoms lasted from 5 to 8 h. Necrosis at 10–30% concentrations occurred for less than 24 h. Necrosis symptoms were seen at 5% concentration but 2.5% of metabolites could not induce chlorosis or necrosis (Fig. 1 F1–F10).

The infiltrated metabolites were from 2 ml extracts of 50 ml culture medium, then the actual concentration of metabolites in the primary culture medium was equal to = 0/0625, i.e., if the medium is diluted 2–1025/6, then the produced metabolites cannot produce necrosis.

### 3.4 TLC and HPLC analysis

TLC technique partially separated the ingredients of the metabolites to 5–7 compounds, of which at least five compounds caused necrosis on *Pelargonium*. AOH and AME showed blue fluorescence under long-wavelength UV light.

The metabolites of three isolates (W19, Z81 and H4) were analyzed by HPLC. The difference between these three isolates was the incidence of necrosis symptoms. W19 caused necrosis in less than 24 h. Z81 had slow degradation rate and the duration of necrosis was less than W19, but its necrosis induction was higher than the other isolates. H4 isolate, which caused necrosis on tomato tissue after 1 week, did not affect *Pelargonium*.

**Fig. 2:**
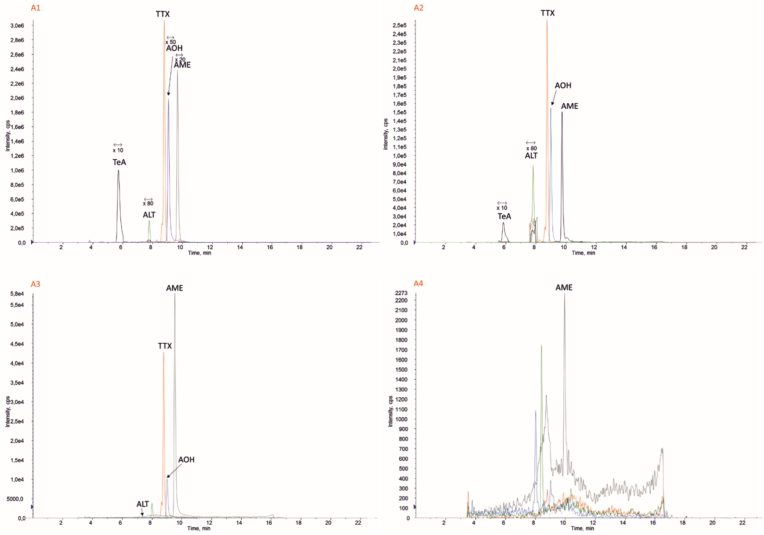
HPLC chromatogram showing *Alternaria* toxin in A1(*A. alternata* W9), A2 (*A. tennuisima* H4), A3 (*Alternaria* sp. Z81) and A4 (Control) metabolites extracted from Czapeck agar.

As seen from the peak areas in the HPLC chromatograms (Fig. 2), five peaks were detected in the injected fungal extract. Based on *Alternaria* mycotoxins standard, this peak were TeA (RT ∼6 min), ALT (RT ∼8 min), TTX (RT ∼8.5–9 min), AOH (RT ∼9.5 min) and AME (RT ∼10 min) toxins. The concentration of each toxin in the injected extract of W19 isolate into HPLC, TeA was about 102.03 ppm, Z81 isolate was 58.3 ppm, 0 for H4 isolate and control. AOH toxin in the W19 isolate was 24.01 ppm, 74.63 ppm in Z81, and 12.61 ppm in the H4 isolate (Table 2).

**Table 2:** Mycotoxin concentration in tne *Alternaria* metabolites (Extract of 50 ml Czapeck media in 2 ml distilled water).

Sample	Isolate	Preliminary injection	ng/ml				
			TEA	AOH	ALT	TTX	AME
A1	W19	Pelargonium necrosis less than 24h	03/102	01/24	04/6	24/959	44/2
A2	Z81	Pelargonium necrosis after one week	58/3	63/74	89/1	04/77	99/2
A3	H4	Tomato necrosis after one week, Pelargonium Not affected	nd	61/12	91/0	59/12	15/1
A4	Czapek	**-**	nd	nd	nd	nd	nd

The ALT toxin produced by W19, Z81, and H4 isolates were 6.04, 1.89, and 0.91 ppm. TTX levels differed in these isolates, 959.24, 77.04, and 12.69 ppm, respectively. AME amounts did not vary significantly and were 2.44, 2.99, and 1.15 ppm (Table 2).

HPLC data in Table 2 shows the amount of *Alternaria* toxins in 2 ml metabolite extracted by chloroform from 50 ml of Czapek culture medium containing *A. alternata* fungi. Therefore, to calculate the concentration of toxins in the original medium, the data in this table should be multiplied by 25 to determine the actual number of these toxins in the culture media.

### 3.5 Pathogenicity test

W19 isolate on *Pelargonium* leaf caused rapid infection development, complete necrosis and leaves death. Z81 isolate caused necrosis around the infiltration site 4 days after inoculation. H4 isolate also caused necrosis, as with Z81 isolate, but the tissue degradation severity was greater than Z81 isolate. In the control sample, which was inoculated with the culture medium, no changes was observed in the tissue (Fig. 1A–B).

The type of symptom development was the same in all three isolates tested on the leaf, but in the W19 isolate, the symptom development rate was higher in the leaf area, but in the other isolates, there was no significant difference in symptom incidence.

## 4 Discussion and conclusion

The results showed that *A. alternata* (W19 isolate) had pathogenicity on *Pelargonium* detached leaves. The metabolites of this isolate contained five different toxins, which was significantly higher than the levels of these toxins in other isolates of *A. alternata* and *A. tenuissima* species. This study is the first report of the presence of these toxins in these two species of *Alternaria* from Iran.

The difference in toxin levels did not affect the pathogenicity rate because these toxins did not have any effects on pathogenicity, or could not produce enough toxins that affect pathogenicity. Therefore, it seems that the amount of toxin production doesn't affect the spread of fungi within the plant and the development of the disease.

The effect of W19 metabolites on a wide range of plants has shown that this toxins can affect the leaf cells’ activities, leading to chlorosis or necrosis, but had little effect on pathogenicity in the leaf.

TeA level in the W19 isolate was about 34 times that of the Z81 isolate. The highest production of AOH occurred in the Z81 isolate, which was three times that of the W19 isolate and six times that of the H4 isolate. The level of ALT in the W19 isolate was approximately three times higher than the Z81 isolate and six times higher than the H4 isolate. W19 produced TTX toxin more than 12 times Z81 isolate and 76 times H4 isolate. AME levels were not significantly different (2.5 ppm). TeA and TTX have phytotoxic activity of fungal extracts, and ALT is highly phytotoxic.

According to Table 1, due to the different times of symptoms incidence, the amount of these three different toxins is also differ, and as in W19 isolate, the appearance of the symptoms is rapid, the amount of these metabolites is also higher, and is less or absent in H4 isolate, which showed no symptoms in *Pelargonium*. The rate of necrosis in plant leaves was much faster than nontoxic isolates. It seems that the concentrations of these toxins and their synergitic effects cause this phenomenon.

Tentoxins produced by *A. alternata* are known as a phytotoxic and induce chlorosis in some plants (Fontaine et al., 2021; Woo et al., 2022). Cucumber cotyledons are very sensitive to TTX, however, this toxin in cucumber does not inhibit the conversion of protochlorophyllide to chlorophyll, but the concentration of chlorophyll is reduced (Halloin et al., 1970). Saad et al. (1970) showed that TTX was active at low concentrations, for example 0.2 μg/ml, in germinated cucumber seedlings under continuous light. The most important effect of TTX may be on by binding to factor 1 (CF1) of chloroplast (Wang et al., 2022).

TeA is also another toxin identified in this study. Different species of *Alternaria* produce this toxin (Gonçalves et al., 2022) which is toxic to a wide range of plants, fungi, bacteria, and viruses, and inhibits eukaryotic protein biosynthesis (Aichinger et al., 2021). TeA inhibits seed germination and reduces seedling growth (Woo et al., 2022).

AME toxin is another toxin identified in this study that causes chlorosis in tobacco leaves. AOH and AME were the main mycotoxins in tomatoes and apples after inoculating with *A. alternata*. Altertoxins I, II, and III are mutagens in *Salmonella typhimurium*. Among these three toxins, Altertoxin III has the highest mutagenicity effect (Dall’Asta et al., 2014; Wang et al., 2022).

In this study, five mycotoxins (TTX, TeA, ALT, AOH, and AME) were detected in *A. alternata* (W19) and *A. tennuisima* isolates and the infiltration of these toxins into a wide range of plants that caused tissue necrosis. The production of each of these toxins and its effects on the plant have been proven by other researchers so far.

## Ethical statement for solid state ionics

Hereby, I/insert author name/consciously assure that for the manuscript/insert title/the following is fulfilled:

1) This material is the authors' own original work, which has not been previously published elsewhere.

2) The paper is not currently being considered for publication elsewhere.

3) The paper reflects the authors' own research and analysis in a truthful and complete manner.

4) The paper properly credits the meaningful contributions of co-authors and co-researchers.

5) The results are appropriately placed in the context of prior and existing research.

6) All sources used are properly disclosed (correct citation). Literally copying of text must be indicated as such by using quotation marks and giving proper reference.

7) All authors have been personally and actively involved in substantial work leading to the paper, and will take public responsibility for its content.

The violation of the Ethical Statement rules may result in severe consequences.

To verify originality, your article may be checked by the originality detection software iThenticate. See also http://www.elsevier.com/editors/plagdetect.

I agree with the above statements and declare that this submission follows the policies of Solid State Ionics as outlined in the Guide for Authors and in the Ethical Statement.

## CRediT authorship contribution statement

**Atefeh Sedighi:** Writing – original draft, Software, Methodology, Investigation, Funding acquisition, Formal analysis. **Abbas Mohammadi:** Writing – review & editing, Writing – original draft, Visualization, Validation, Supervision, Software, Resources, Project administration, Methodology, Investigation, Funding acquisition, Formal analysis, Data curation, Conceptualization.

## Declaration of competing interest

The authors declare that they have no known competing financial interests or personal relationships that could have appeared to influence the work reported in this paper.

## Data Availability

No data was used for the research described in the article.
